# Simvastatin inhibits TGFβ1-induced fibronectin in human airway fibroblasts

**DOI:** 10.1186/1465-9921-12-113

**Published:** 2011-08-24

**Authors:** Dedmer Schaafsma, Karol D McNeill, Mark M Mutawe, Saeid Ghavami, Helmut Unruh, Eric Jacques, Michel Laviolette, Jamila Chakir, Andrew J Halayko

**Affiliations:** 1 Departments of Physiology & Internal Medicine, and Section of Respiratory Disease, University of Manitoba, Winnipeg, MB, Canada; 2 Biology of Breathing Theme, Manitoba Institute of Child Health, Winnipeg, MB, Canada; 3 CIHR IMPACT Training Program in Pulmonary and Cardiovascular Research, University of Manitoba, Winnipeg, MB, Canada; 4 CIHR National Training Program in Allergy and Asthma, University of Manitoba, Winnipeg, MB, Canada; 5 Section of Thoracic Surgery, University of Manitoba, Winnipeg, MB, Canada; 6 Institut Universitaire de Cardiologie et de Pneumologie de Québec, Québec, Canada

**Keywords:** airway fibroblasts, airway remodeling, asthma, fibronectin, geranylgeranyl transferase, statins

## Abstract

**Background:**

Bronchial fibroblasts contribute to airway remodelling, including airway wall fibrosis. Transforming growth factor (TGF)-β1 plays a major role in this process. We previously revealed the importance of the mevalonate cascade in the fibrotic response of human airway smooth muscle cells. We now investigate mevalonate cascade-associated signaling in TGFβ1-induced fibronectin expression by bronchial fibroblasts from non-asthmatic and asthmatic subjects.

**Methods:**

We used simvastatin (1-15 μM) to inhibit 3-hydroxy-3-methlyglutaryl-coenzyme A (HMG-CoA) reductase which converts HMG-CoA to mevalonate. Selective inhibitors of geranylgeranyl transferase-1 (GGT1; GGTI-286, 10 μM) and farnesyl transferase (FT; FTI-277, 10 μM) were used to determine whether GGT1 and FT contribute to TGFβ1-induced fibronectin expression. In addition, we studied the effects of co-incubation with simvastatin and mevalonate (1 mM), geranylgeranylpyrophosphate (30 μM) or farnesylpyrophosphate (30 μM).

**Results:**

Immunoblotting revealed concentration-dependent simvastatin inhibition of TGFβ1 (2.5 ng/ml, 48 h)-induced fibronectin. This was prevented by exogenous mevalonate, or isoprenoids (geranylgeranylpyrophosphate or farnesylpyrophosphate). The effects of simvastatin were mimicked by GGTI-286, but not FTI-277, suggesting fundamental involvement of GGT1 in TGFβ1-induced signaling. Asthmatic fibroblasts exhibited greater TGFβ1-induced fibronectin expression compared to non-asthmatic cells; this enhanced response was effectively reduced by simvastatin.

**Conclusions:**

We conclude that TGFβ1-induced fibronectin expression in airway fibroblasts relies on activity of GGT1 and availability of isoprenoids. Our results suggest that targeting regulators of isoprenoid-dependent signaling holds promise for treating airway wall fibrosis.

## Background

Chronic obstructive airways diseases, including asthma and COPD, are characterized by structural alterations of the airway wall. The accumulation of extracellular matrix (ECM) proteins (fibrosis) and augmentation of the airway mesenchymal layer, including fibroblasts and airway smooth muscle, are common features of this airway remodeling [1-3]. In asthma, the degree of subepithelial fibrosis has been shown to be associated with disease severity and correlated with a decline in lung function parameters [4]. Transforming growth factor β1 (TGFβ1) is a principal mediator of subepithelial fibrosis and is highly expressed in asthmatics [4-6]. Airway fibroblasts and myofibroblasts are a primary source of ECM proteins, including fibronectin, in subepithelial fibrosis linked to airway remodeling [7]. Targeting and understanding molecular mechanisms that drive the pro-fibrotic potential of these cells is of great interest with respect to the development of therapies for chronic airways diseases.

Statins were initially developed to inhibit the activity of 3-hydroxy-3-methylglutaryl-coenzyme A (HMG-CoA) reductase and are widely prescribed to reduce hyperlipidemia [8]. Substantial evidence shows that statins also have pleiotropic anti-inflammatory, anti-fibroproliferative and immunomodulatory effects that are independent of their cholesterol-lowering capacity [9-14]. HMG-CoA reductase is the proximal rate-limiting enzyme of the multistep mevalonate cascade for cholesterol biosynthesis. Cholesterol intermediates include the 15- and 20-carbon isoprenoids, farnesylpyrophosphate (FPP) and geranylgeranylpyrophosphate (GGPP), respectively. These lipid moieties are substrates for farnesyl transferase (FT) and geranylgeranyl transferase 1 (GGT1) that catalyze the modification of monomeric G-proteins, such as Ras and RhoA, by conjugating lipid anchors crucial for their association with and activation at the plasma membrane. Effects of statins on cell physiology have been attributed, in part, to the depletion of isoprenoids and the ensuing effects on prenylation-dependent intracellular signaling activity [15-18]. Given the biological importance of FT and GGT1, a number of selective inhibitors have been developed and tested in clinical trials for treatment of cancer [19-21]. To date the impact of these inhibitors on lung health has not been established.

In previous work, we showed that mevalonate-derived isoprenoids provide key regulatory input for the fibrotic response of human airway smooth muscle cells [14]. We now investigate the role of mevalonate cascade-associated cell signaling in TGFβ1-induced expression of the extracellular matrix protein fibronectin by bronchial fibroblasts from both non-asthmatic and asthmatic subjects.

## Materials and methods

### Materials

All chemicals were obtained from Sigma (St. Louis, MO) unless indicated otherwise. Primary antibodies against fibronectin (sc-9068, rabbit polyclonal), collagen type I (sc-8786, goat polyclonal), GGTase 1β (sc-100820, mouse monoclonal) and FT β (sc-137, rabbit polyclonal) were from Santa Cruz Biotechnology (Santa Cruz, CA).

### Human airway fibroblast cell culture: standard study design

**Figure 1:**
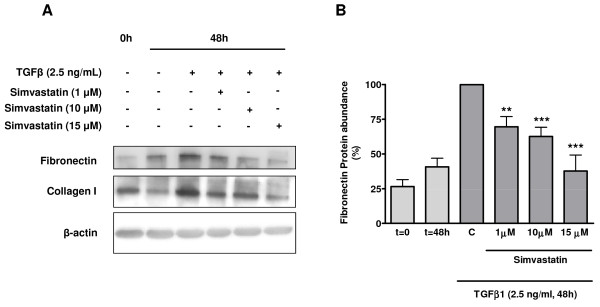
**Simvastatin prevents TGFβ1-induced fibronectin and collagen I expression**. Western analysis was performed using whole cell lysates from primary human airway fibroblast cultures that were grown to ~80% confluence, then serum-deprived for 24 h and stimulated with TGFβ1 (2.5 ng/ml) for 48 hours. A typical blot is shown for fibronectin and collagen I (A) alongside data from densitometry analyses, which revealed that TGFβ1-induced fibronectin protein abundance was dose-dependently suppressed by simvastatin (1-15 μM). For each experiment expression levels were normalized to maximal-induced fibronectin expression, and β-actin was used as a loading control. Data represent means ± s.e. mean of 3 independent experiments, using 3 different primary cell lines. **P < 0.01, ***P < 0.001 compared to C. *Legend*: 0 h and t = 0: initial basal (unstimulated) cultures; 48 h and t = 48 h: treatment time-matched, basal (unstimulated) cultures; C: cells treated with TGFβ1 alone for 48 hours.

**Figure 2:**
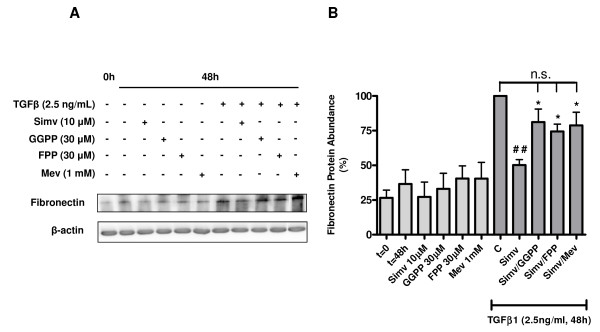
**Involvement of isoprenoid intermediates of the mevalonate cascade in the simvastatin-induced suppression of TGFβ1-induced fibronectin expression**. (A) Representative western blot showing fibronectin protein abundance in whole cell lysates obtained from primary human airway fibroblast cultures stimulated with TGFβ1 (2.5 ng/ml) in the presence or absence of simvastatin (10 μM) and co-incubated with GGPP (30 μM), FPP (30 μM) or mevalonate (1 mM) for 48 h. (B) Data from densitometry of samples from experiments shown in panel A. Data represent means ± s.e. mean of 3 independent experiments, using 3 different cell lines. *Legend: *0 h and t = 0: initial basal (unstimulated) cultures; 48 h and t = 48 h: treatment time-matched, basal (unstimulated) cultures; C: cells treated with TGFβ1 alone for 48 hours; Simv: simvastatin; Mev: mevalonate. ^##^P < 0.01 compared to C(ontrol); *P < 0.05 compared to simvastatin.

**Figure 3:**
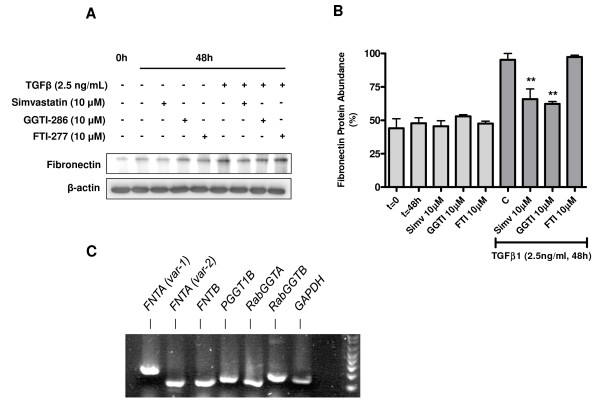
**GGTI-286 mimics the effects of simvastatin on TGFβ1-induced fibronectin protein expression**. Western analysis was performed using whole cell lysates obtained from primary human airway fibroblast cultures that were maintained under TGFβ1 (2.5 ng/ml)-stimulated or serum-deprived conditions in the absence or presence of simvastatin (10 μM), GGTI-286 (10 μM) or FTI-277 (10 μM) for 48 h. (A) Representative western blots showing that the suppressive effects of simvastatin on TGFβ1-induced fibronectin protein expression could be mimicked by GGTI-286, but not by FTI-277. (B) Bar chart summarizing the effects of simvastatin, GGTI-286 and FTI-277 under basal and TGFβ1-stimulated conditions on fibronectin protein abundance. (C) Reverse transcriptase PCR was performed using total RNA extracted from human fibroblast cell cultures. The photograph shows that transcripts for 7 prenyl transferase subunits are abundant in these cells, including multiple variants of the FNTA subunit that is common to GGT1 and FT. Data in panel B represent means ± s.e. mean of 3 independent experiments, using 3 different cell lines. *Legend: *0 h and t = 0: initial basal (unstimulated) cultures; 48 h and t = 48 h: treatment time-matched, basal (unstimulated) cultures; C: cells treated with TGFβ1 alone for 48 hours; Simv: simvastatin. **P < 0.01 compared to C(ontrol).

**Figure 4:**
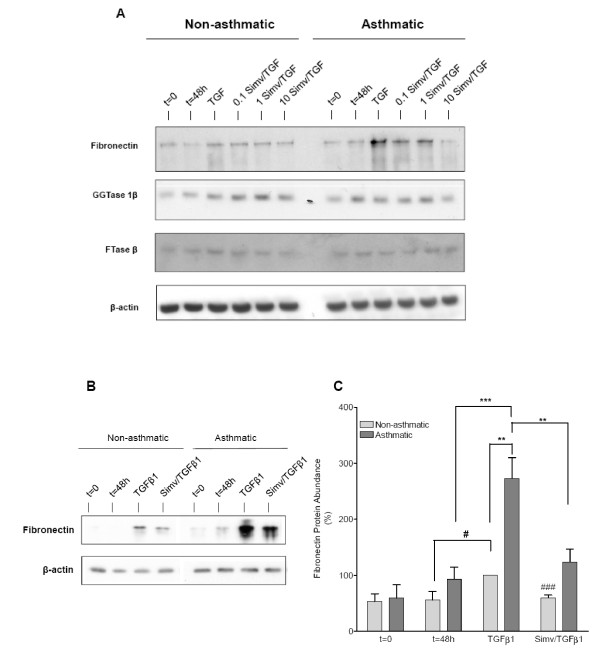
**Simvastatin profoundly suppresses the augmented TGFβ1-induced fibronectin expression in asthmatic bronchial fibroblasts**. Western analysis was performed using whole cell lysates obtained from nonasthmatic and asthmatic bronchial fibroblast cultures that were maintained under TGFβ1 (2.5 ng/ml)-stimulated or serum-deprived conditions in the absence or presence of simvastatin (0.1-10 μM) for 48 h. (A) Western blots showing simvastatin dose-dependently suppresses TGFβ1-induced fibronectin expression in nonasthmatic and asthmatic bronchial fibroblasts, whereas GGTase 1β and FTase β protein abundance is not affected. (B) Representative western blot showing a more robust TGFβ1-induced fibronectin abundance in asthmatic versus nonasthmatic fibroblasts, which is effectively suppressed by simvastatin. (C) Densitometric analysis revealed significant differences in TGFβ1-induced fibronectin expression between nonasthmatic and asthmatic fibroblasts. Moreover, simvastatin markedly prevents fibronectin expression in both cell types. Data represent means ± s.e. mean of 5 independent experiments, using cells obtained from 3 non-asthmatic and 3 asthmatic subjects. *Legend: *t = 0: initial basal (unstimulated) cultures; t = 48 h: treatment time-matched, basal (unstimulated) cultures; Simv: simvastatin. **P < 0.01, ***P < 0.001 compared to asthmatic TGFβ. ^#^P < 0.05, ^###^P < 0.001 compared to nonasthmatic TGFβ.

Primary human airway fibroblasts were isolated from macroscopically healthy segments of second- to fourth-generation main bronchi obtained after lung resection surgery from patients with a diagnosis of adenocarcinoma. The airway smooth muscle and mesenchymal fibroblast layers were carefully separated by manual dissection; passage 3-4 fibroblasts were used (Figures 1, 2, and 3). For comparative studies (Figure 4) primary fibroblasts were isolated from bronchial biopsies of mild steroid naïve asthmatic (n = 3) and healthy (n = 3) subjects. The asthmatic subjects fulfilling the American Thoracic Society criteria for asthma [22] were recruited from the Asthma Clinic at IUCPQ (Québec, Canada). They used only an inhaled β2-agonist on demand. The asthmatics were atopic nonsmokers (mean age = 24 ± 2, FEV_1_% predicted = 95 ± 0.4% and PC_20 _= 4.6 ± 0.01 mg/ml). None used systemic or inhaled CS. Healthy subjects (mean age = 22 ± 0.4, FEV_1_% predicted = 106 ± 0.82% and PC_20 _≥ 128 mg/ml) were non-atopic nonsmokers with no history of asthma or other pulmonary or systemic diseases. The atopic status of asthmatics was determined by skin prick tests showing a positive reaction (3 mm or more) to at least 2 aero-allergens. The healthy group had no skin reaction. Bronchial biopsies were obtained by bronchoscopy from asthmatic and healthy subjects as described previously [23]; passage 4-6 cells were used. Written informed consent was obtained from all subjects before entry into the study. All procedures were approved by the Human Research Ethics Board (University of Manitoba) and the Ethics Committee at the Institut Universitaire de Cardiologie et de Pneumologie de Québec.

Cells were plated on uncoated plastic dishes in Dulbecco's modified Eagle's medium (DMEM) supplemented with 50 U/ml streptomycin, 50 μg/ml penicillin, and 10% fetal bovine serum (FBS). Cells were grown to ~80% confluence, after which they were maintained for 24 hours in serum-free DMEM supplemented with 5 μg/ml insulin, 5 μg/ml transferrin, and 5 ng/ml selenium.

For all studies, unless otherwise stated, we followed a standard treatment protocol. Serum-deprived cells were stimulated with TGFβ1 (2.5 ng/ml) for 48 hrs in the presence or absence of simvastatin (1, 10, 15 μM, added 30 min before TGFβ1). In some experiments, the effects of co-incubation with mevalonic acid (1 mM) [14,24], geranylgeranyl pyrophosphate (GGPP, 30 μM) [14,18] or farnesyl pyrophosphate (FPP, 30 μM) [14,18] were studied (all added 4 h prior to TGFβ1). In separate experiments the effects of the geranylgeranyltransferase inhibitor GGTI-286 (10 μM) [14,18,24,25] and the farnesyltranferase inhibitor FTI-277 (10 μM) [14,25,26] were investigated (added 30 min prior to TGFβ1).

### Protein immunoblotting

After washing cultures with ice-cold phosphate-buffered saline (PBS, composition (mM): NaCl 140.0; KCl 2.6; KH_2_PO_4 _1.4; Na_2_HPO_4_.2H_2_O 8.1; pH 7.4) cell lysates were prepared in ice-cold SDS buffer (composition: 62.5 mM Tris-HCl, 2% SDS, 1 mM PMSF, 1 mM protease inhibitor mix, and 1 mM phosphatase inhibitor mix). Equal amounts of protein, as determined using a commercial Lowry assay, were subjected to electrophoresis and transferred to nitrocellulose membranes. Membranes were subsequently blocked in Tris buffer containing 0.1% Tween-20 and 5% w/v dried milk powder, then incubated overnight at 4°C with primary antibodies (fibronectin (diluted 1:1000), GGTase 1β (diluted 1:400), FTβ (diluted 1:1000) and β-actin (diluted 1:20.000)). Blots were then incubated with diluted horseradish peroxidase conjugated secondary antibodies prior to visualizing bands on film using enhanced chemiluminescence reagents (Amersham, Buckinghamshire, UK). Al blots were subjected to densitometry using a computer page scanner and Totallab™software. For data analyses bands were normalized to β-actin to correct for small differences in loading.

### RNA extraction and reverse transcriptase PCR

Total RNA was extracted using the RNeasy RNA Mini Kit (Qiagen, U.S.A). For reverse transcription (first strand cDNA synthesis) we used 2 μg of total RNA (in × μL), 0.3 μL Random Hexamers (3 mg/mL, Invitrogen) and 10-x μL ddH_2_O. After heating for 5 min at 65°C, 9 μL of reaction mixture (1 μL dNTP PCR mix (10 mM, Amersham, Canada), 4 μL 5 × first-strand buffer, 2 μL DTT (0.1 M), 1 μL RNaseOUT (40 U) and 1 μL Moloney murine leukemia virus reverse transcriptase (M-MLV RT, 200 U, Invitrogen)) was added. Samples were incubated at 42°C for 120 minutes then heating at 72°C for 15 minutes. cDNA was stored at -20°C until further use. PCR amplification was performed in a total volume of 50 μL which included 1 μL RT reaction mixture, 0.5 μM of each forward and reverse oligonucleotide, 1 × PCR buffer with 1.5 mM MgCl, 0.2 mM dNTP PCR mix and 1.25 U of Platinum Taq Polymerase (Invitrogen). Primers used for GAPDH and the human prenyltransferase subunits FNTA (var1), FNTA (var2), FNTB, PGGT1B, RabGGTA and RabGGTB are listed in Table 1.

**Table 1:** Primers Used For Reverse Transcriptase PCR

Fragment	Forward	Reverse	PCR Product Size
FNTA var 1(Human)	5'-TAT AGA TCC GGT GCC GCA GAA TGA-3'	5'-ACT CTC CGG AAA TGC CAC ACT GTA-3'	196 bp

FNTA var 2 (Human)	5'-GTC CTG CAG CGT GAT GAA AGA AGT-3'	5'-ACT CTC CGG AAA TGC CAC ACT GTA-3'	101 bp

FNTB (Human)	5'-TGC AGA GGG AGA AGC ACT TCC ATT-3'	5'-AGC TGT GCA GGA TCC AAT AGC AGA-3'	114 bp

PGGT1B (Human)	5'-TTG CAA TGA CCT ACA CTG GCC TCT-3'	5'-TCA CTG CCT TCA GGT ACT GCA CAA-3'	143 bp

RabGGTA (Human)	5'-TGC TGG AGA ATA GCG TGC TCA AGA-3'	5'-AGT CAA GAT GGG TGA CCA AGA GCA-3'	121 bp

RabGGTB (Human)	5'-AGA CCA GGT TCT GAA TCC CAT GCT-3'	5'-TGG TAA CTT CTC CGG CCT TCC ATT-3'	162 bp

GAPDH (Human)	5'-AGC AAT GCC TCC TGC ACC ACC AAC-3'	5'-CCG GAG GGG CCA TCC ACA GTC T-3'	136 bp

### Statistical analysis

All data represent means ± s.e. mean from n separate experiments. Statistical significance of differences was evaluated by the Student's *t*-test for paired observations or by ANOVA for multiple measurements followed by a Tukey's post-test. Differences were considered to be statistically significant when *P *< 0.05.

## Results

### Simvastatin prevents TGFβ1-induced fibronectin protein expression

Primary human bronchial mesenchymal fibroblasts were stimulated with 2.5 ng/ml TGFβ1 for 48 h in the presence and absence of simvastatin (Figures 1A and 1B). TGFβ1 induced a marked increase in fibronectin protein, an effect significantly suppressed by 1 (69.5 ± 7.4% of C), 10 (62.5 ± 6.7% of C) and 15 μM (37.6 ± 11.5% of C) simvastatin. Similarly, TGFβ1-induced collagen I protein abundance was dose-dependently inhibited by simvastatin (Figure 1A), indicating that as for airway smooth muscle [14] the inhibitory effects of simvastatin are more broadly applicable. Based on these data and previous reports by our group on potential toxicity of high concentrations of simvastatin [27], we used 10 μM in all subsequent experiments.

### Depletion of isoprenoids underpins the suppressive effects of simvastatin

To determine whether the effects of simvastatin on fibronectin are due to reduced formation of mevalonate, FPP and GGPP, we incubated human airway fibroblasts with TGFβ1 and simvastatin in the presence of mevalonate (1 mM), FPP (30 μM) or GGPP (30 μM). Co-incubation with these intermediates caused nearly full prevention of the suppressive effects of simvastatin, implying their depletion is critical for the effects of simvastatin (Figures 2A and 2B).

### Inhibition of GGT1, but not FT, mimics the effects of simvastatin

We next investigated the effects of the geranylgeranyl transferase inhibitor GGTI-286 (10 μM) and the farnesyl transferase inhibitor FTI-277 (10 μM) on TGFβ1-induced fibronectin protein expression (Figures 3A and 3B). GGTI-286 significantly prevented TGFβ1-induced fibronectin accumulation to a similar degree as 10 μM simvastatin. In contrast, no reduction in fibronectin was observed after co-treatment with FTI-277 (Figures 3A and 3B). These findings indicate a predominant involvement of GGT1, but not FT, in the TGFβ1-induced profibrotic response of human airway fibroblasts. In line with these findings, profiling of the expression of protein prenyltransferase subunits by RT-PCR revealed expression of 6 subunits, including two variants of the farnesyltranferase, CAAX box, alpha (FNTA) subunit that is common to both GGT1 and FT (Figure 3C). These results indicate human airway fibroblasts express the genes necessary to form GGT1, FT and GGT2 prenyltransferase heterodimers. Further confirming these findings, we demonstrate that GGTase 1β and FTase β protein are expressed in non-asthmatic and asthmatic fibroblasts; abundance of these subunits was not affected by simvastatin, nor was there any difference in expression between non-asthmatic and asthmatic fibroblasts (Figure 4A).

### Simvastatin effectively suppresses the augmented profibrotic response of asthmatic bronchial fibroblasts

To determine the effects of simvastatin on fibronectin expression in non-asthmatic and asthmatic bronchial fibroblasts, cells were stimulated with TGFβ1 in the presence and absence of simvastatin (Figure 4A). Simvastatin dose-dependently suppressed fibronectin abundance in non-asthmatic and asthmatic fibroblasts. To directly compare these responses different lysates were analyzed on the same gel; though no differences in basal fibronectin expression were observed, a more robust response to TGFβ1 by asthmatic fibroblasts was evident (2.7 ± 0.4 fold higher; Figures 4B and 4C). Importantly, simvastatin suppressed TGFβ1-induced fibronectin expression in both non-asthmatic and asthmatic cells (40.0 ± 4.8% and 52.4 ± 8.1% reduction, respectively; Figure 4C).

## Discussion

In the present study, we demonstrate that isoprenoid intermediates of the mevalonate cascade provide key regulatory input for the TGFβ1-induced expression of the extracellular matrix protein fibronectin by human bronchial fibroblasts. HMG-CoA reductase inhibition with simvastatin suppressed TGFβ1-induced fibronectin abundance, an effect prevented by exogenous mevalonate, GGPP and FPP. Effects of simvastatin were mirrored by the selective GGT1 inhibitor, GGTI-286, but not the farnesyl protein transferase inhibitor, FTI-277, suggesting that proteins targeted by GGT1 for conjugation of prenyl lipid chains are crucial for TGFβ1-induced fibronectin expression. Moreover, we show for the first time that fibronectin expression in response to TGFβ1 is markedly augmented in bronchial fibroblasts obtained from asthmatics compared to those from non-asthmatics. Simvastatin effectively inhibited TGFβ1-induced fibronectin in fibroblasts from both groups.

Statins are recognized for pleiotropic effects that exceed their cholesterol lowering capacity. Statin use correlates with reduced COPD hospitalizations and mortality [28-30], and up to 50% slower decline in lung function (FEV1 and FVC) in smokers, former smokers and non-smokers [9,10]. In patients receiving double lung transplant, statin use is associated with significantly better post-operative spirometry and airway inflammation as indicated by reduced numbers of neutrophils and lymphocytes [31]. Several recent studies have also revealed anti-inflammatory effects of statins in murine and rat models of allergic asthma [32,33] and COPD [11,12]. Moreover, statins reportedly suppress *ex vivo *airway responsiveness in animal models [34,35].

Statins have broad effects on cell responses, including inhibition of proliferation, migration and they can promote apoptosis [15-18,27]. These studies are consistent with our observation that mevalonate, GGPP and FPP can prevent the effects of simvastatin, confirming the fundamental role of regulated protein lipidation in cell function, including fibronectin expression [36]. Importantly, we have demonstrated previously that under the conditions studied 10 μM simvastatin does not affect human airway fibroblast viability, as determined by MTT assays, within 48 h; indicating the observed decrease in fibronectin is not an artifact due to cell death [27]. Our finding that mevalonate, FPP and GGPP prevent the suppressive effects of simvastatin yet only GGTI-286, but not FTI-277, mimics its actions suggests that signaling proteins that are subject to GGT1-catalyzed geranylgeranylation are crucial for TGFβ1-induced fibronectin expression in airway fibroblasts. These findings are supported by studies using human fetal lung fibroblasts demonstrating the effectiveness of a GGT1 inhibitor (GGTI-298), but not a FT inhibitor (FTI-277), on TGFβ1-mediated expression of connective tissue growth factor (CTGF), elastin and fibronectin mRNA [21,37-39].

The lack of effect of FT inhibition versus the effectiveness of FPP to prevent the inhibitory effects of simvastatin seems paradoxical. Theoretically, FPP can be converted to GGPP intracellular, as such providing a substrate for GGT1 [40]. Although an interesting hypothesis, in the presence of simvastatin, even with the addition of FPP, formation of the more downstream sterol intermediate GGPP is not effected as HMG-CoA inhibition depletes the upstream 5-carbon upstream intermediate, isopentyl pyrophosphate (IPP), that is required for conversion of FPP to GGPP [40]. An alternative potential explanation could lie in the promiscuity of GGT1 both in using alternate isoprenoids (i.e. FPP) and in effectively prenylating downstream effectors that are essential for TGFβ1-induced fibronectin expression. Thus, our findings suggest that GGT1 may be able to utilize FPP to modify a critical downstream effector. Moreover, we speculate that FT is unable to prenylate signaling proteins and induce their activation when GGT1 activity is suppressed with GGTI-286. These complex topics need to be addressed mechanistically in future studies.

The anti-fibrotic effects of statins are not likely to be limited to airway mesenchymal cells. Indeed, beneficial effects of statins on human hypertrophic cardiomyopathy [41] and the occurrence of renal interstitial fibrosis in transgenic rabbits [42] have been reported. In addition, statins have cardioprotective effects that are associated with their anti-fibrotic effects in adrenomedulin-knockout mice [43] and have been reported to prevent left ventricular remodelling, including interstitial fibrosis, in hypertensive rats [44]. *In vitro *studies using human lung fibroblasts derived from healthy and idiopathic pulmonary fibrosis (IPF) patients also demonstrate that simvastatin can inhibit connective tissue growth factor expression, reduce collagen gel contraction, and down-regulate smooth muscle α-actin expression [45]. In addition, systemic administration of simvastatin markedly attenuates the onset of collagen-associated lung fibrosis in mice treated with trachea-instilled bleomycin [46].

To our knowledge, we demonstrate for the first time that TGFβ1-induced fibronectin protein expression is significantly greater in fibroblasts from asthmatic subjects compared to those obtained from healthy subjects. These results correlate well with findings by Westergren-Thorsson and colleagues that demonstrate fibroblasts isolated from asthmatics produce increased amounts of proteoglycans [47]. This intrinsic difference between asthmatic and non-asthmatic fibroblasts to express ECM proteins could contribute to sub-epithelial fibrosis in the asthmatic airway. Our data indicate that fibronectin expression by asthmatic fibroblasts is not-refractory to simvastatin, suggesting this therapeutic approach could be of benefit. In clinical studies, short-term treatment of asthmatics with statins had no significant effect on lung function or other indices of asthma control in patients treated with corticosteroids [48] or without anti-inflammatory medication [49]. Conversely, a recent study revealed that simvastatin can enhance the anti-inflammatory effects of inhaled corticosteroids in mild asthmatics [50], which is in line with reduced alveolar macrophage numbers in sputum of asthmatics that had received statin treatment [48]. Inasmuch as these studies indicate that the effects of short-term statin treatment on airway inflammation and lung function in mild to moderate asthmatics is debatable, the effects of statins on features of airway remodelling, which are generally associated with disease duration and severity, remain elusive. Recent in vitro studies using human airway smooth muscle cells and fibroblasts do show statins inhibit proliferation and promote apoptosis [18,51], which when considered in the context of previous work by our group [14] and the present study showing a concomitant effect on fibronectin expression in bronchial mesenchymal cells, suggests potential for suppressing airway remodeling.

## Conclusions

Our data indicate that mevalonate cascade associated cell signaling is a key signaling component in TGFβ1-induced fibronectin expression in primary human airway fibroblasts. Moreover, it appears that the prenyltransferase GGT1 is a principal effector for isoprenoid-dependent TGFβ1 induced fibronectin expression. Last, we demonstrate the presence of exaggerated fibronectin expression in response to TGFβ1 in asthmatic fibroblasts, and confirm that simvastatin can significantly suppress the response in these cells. Based on our results simvastatin and perhaps more selective inhibitors of GGT1 could be considered as potential therapeutic tools to modulate airway wall fibrosis in fibrotic airway diseases such as asthma.

## Abbreviations

ECM: extracellular matrix; FPP: farnesylpyrophosphate; FT: farnesyl transferase; GGPP: geranylgeranyl pyrophosphate; GGTase: geranylgeranyl transferase; HMG-CoA: 3-hydroxy-3-methlyglutaryl-coenzyme A; TGF: transforming growth factor.

## Competing interests

The authors declare that they have no competing interests.

## Authors' contributions

DS participated in the design and coordination of the study, performed a major part of the experiments, performed the statistical analysis and drafted the manuscript. KDM, MMM and SG substantially assisted in performing the experiments. EJ, HU, and ML were responsible for tissue collection and handling. JC participated in interpretation of results and the preparation of the manuscript. AJH supervised the study, participated in its design and data interpretation, and was involved in the manuscript preparation. All authors read and approved the final manuscript.
